# Inflammatory Dietary Pattern Predicts Dyslipidemia and Anemia in Middle-Aged and Older Taiwanese Adults with Declined Kidney Function: A Cross-Sectional Population Study from 2008 to 2010

**DOI:** 10.3390/nu11092052

**Published:** 2019-09-02

**Authors:** Adi Lukas Kurniawan, Chien-Yeh Hsu, Hsiao-Hsien Rau, Li-Yin Lin, Jane C-J Chao

**Affiliations:** 1 School of Nutrition and Health Sciences, College of Nutrition, Taipei Medical University, 250 Wu-Hsing Street, Taipei 11031, Taiwan; 2 Department of Information Management, National Taipei University of Nursing and Health Sciences, 365 Ming-Te Road, Peitou District, Taipei 11219, Taiwan; 3 Master Program in Global Health and Development, College of Public Health, Taipei Medical University, 250 Wu-Hsing Street, Taipei 11031, Taiwan; 4 Joint Commission of Taiwan. 31 Sec. 2 Sanmin Road, Banqiao District, New Taipei City 22069, Taiwan; 5 Nutrition Research Center, Taipei Medical University Hospital, 252 Wu-Hsing Street, Taipei 11031, Taiwan

**Keywords:** dietary pattern, inflammation, dyslipidemia, anemia, kidney function, reduced rank regression

## Abstract

Dyslipidemia, anemia, and inflammation are associated with declined kidney function. This study investigated the association of inflammatory dietary pattern with dyslipidemia, anemia, and kidney function biomarkers among middle-aged and older Taiwanese adults with declined kidney function. Biochemical data and food frequency questionnaire were obtained from 41,128 participants with estimated glomerular filtration rate (eGFR) <90 mL/min/1.73 m^2^ and positive urinary protein. Inflammatory dietary pattern was identified by reduced rank regression with C-reactive protein (CRP) and neutrophil-to-lymphocyte ratio (N/L) as response variables. Males had higher prevalence of dyslipidemia and higher inflammatory markers, but lower prevalence of anemia and lower eGFR levels compared to females. Inflammatory dietary pattern characterized with low intakes of seafood, grains, vegetables, and fruits but high intakes of meat, eggs, preserved/processed foods, and sugary drinks was associated with an increased risk of dyslipidemia by 21% in males and an increased risk of anemia by 28–47% in both genders. Furthermore, high consumption of inflammatory dietary pattern was associated with reduced eGFR (males β = −0.85, 95% CI −1.26 to −0.43, females β = −0.53, 95% CI −0.98 to −0.08) and increased N/L and/or CRP in both genders. In conclusion, inflammatory dietary pattern is positively associated with dyslipidemia, anemia, and decreased kidney function in middle-aged and older adults with declined kidney function.

## 1. Introduction

Dyslipidemia is a well-known risk factor for cardiovascular disease (CVD) in the general population. Dysregulation of lipoprotein metabolism in dyslipidemia patients was shown to be associated with a higher risk of developing renal dysfunction [1]. Some clinical data have reported that high triglycerides (TG) is an independent risk factor for chronic kidney disease (CKD) and low high-density lipoprotein cholesterol (HDL-C) can predict CKD progression [2,3,4]. These metabolic alterations may further worsen and contribute to high morbidity and mortality in patients with kidney disease [5]. Additionally, anemia might begin to develop in the early stages of CKD and is commonly observed in more advanced CKD stages [6]. A cross-sectional study using data from the United States National Health and Nutrition Examination Survey in 2007 and 2010 reported that the prevalence of anemia was 15.4% in CKD patients, which represented an estimate of 4.8 million people and it was increased from 8.4% in CKD patients at stage 1 to 53.4% in those at stage 5 [7]. Anemia may initiate or accelerate the development of ventricular mass or left ventricular hypertrophy (LVH) [8]. Previous studies have reported that a combination of moderately declined kidney function, anemia, and LVH was associated with an increased risk of CVD events and mortality [9,10]. Therefore, both dyslipidemia and anemia are important risk factors for the development of cardiovascular events and facilitate renal function deterioration in people with declined kidney function. In addition, chronic low-grade inflammation has been proposed as an underlying pathophysiological mechanism for the development of chronic diseases such as CVD and CKD [11,12]. Inflammation has also been considered one of the main factors in the development of CVD and anemia in CKD patients [13,14]. Serum hepcidin, the predominant hormone of iron homeostasis, is elevated to prevent the use of absorbed iron or stored iron for erythropoiesis in the inflammatory process of chronic disease such as CKD [14]. In this study, we used C-reactive protein (CRP) and neutrophil-to-lymphocyte (N/L) ratio as inflammatory markers. Recently, N/L ratio has been shown to have a relationship with kidney disease and found to be a predictor of cardiovascular events and all-cause mortality [15,16].

Dietary intake is an important modifiable factor associated with developing chronic diseases. Additionally, specific dietary pattern was found to be associated with low-grade inflammation in the previous study [12]. Several studies showed that higher intake of protein, saturated fat, and *trans*-fat from animal food was associated with the progression of declined kidney function and the incidence of CVD [17,18]. Whereas the traditional study of public health nutrition has focused on individual nutrient intake, dietary pattern may be a better solution to examine the interactive influence of diet on the incidence of chronic disease. Dietary patterns are also easier to interpret into practical nutrition education to the population [19]. We hypothesized that dietary pattern plays an important role in the outcome of inflammation-related chronic diseases such as anemia, cardiovascular events, and faster renal function decline. Therefore, the objective of this study was to investigate the relationship of inflammatory dietary pattern with dyslipidemia, anemia, and kidney function biomarkers in the participants with declined kidney function.

## 2. Materials and Methods

### 2.1. Study Participants

A total of 151,206 participants with estimated glomerular filtration rate (eGFR) <90 mL/min/1.73 m^2^ and positive urinary protein were retrieved from the Mei Jau (MJ) Health Institute database from 2008 to 2010. The participants (*n* = 110,078) were excluded due to (1) age <40 years, (2) having any types of cancer, cirrhosis, or virus infection, (3) having a history of kidney surgery, (4) without filling the questionnaire and with missing dietary data, (5) having error results in the biochemical data, or (7) having multiple entries. Finally, 41,128 participants were included in the analysis. The MJ Health Institute is a membership-oriented private institute that provides periodic health examination in four health screening centers (Taipei, Taoyuan, Taichung, and Kaohsiung) in Taiwan. A signed consent form authorized by the MJ Health Institute was collected from all participants before they had a health examination. Joint Institutional Review Board of Taipei Medical University approved this study (TMU-JIRB N201802006).

### 2.2. Clinical and Biochemical Data and Definition of the Diseases

Body mass index (BMI) and blood pressure were measured during the health check-up. Biochemical data including complete blood count (Abbott Cell-Dyn 3700, Abbott Park, IL, USA) such as red blood cells (RBC), hemoglobin, hematocrit, mean corpuscular volume (MCV), mean corpuscular hemoglobin (MCH), mean corpuscular hemoglobin concentration (MCHC), red blood distribution width (RDW), leukocytes, neutrophils, and lymphocytes were measured in each participant. Moreover, C-reactive protein (CRP), fasting blood glucose (FBG), TG, total cholesterol (TC), HDL-C, low-density lipoprotein cholesterol (LDL-C), iron, and blood urea nitrogen (BUN) (Toshiba C8000 auto-analyzer, Tokyo, Japan) were analyzed at the central laboratory of the MJ Health Institute. Uncompensated Jaffe method with alkaline picrate kinetic test was used to measure serum creatinine (CRE) levels. Urinary protein was measured by Roche Miditron M semi-automated computer-assisted urinalysis system. Urinary protein was reported as one or more pluses (+) and eGFR was estimated using the Modification of Diet in Renal Disease Study (MDRD) equation [20]. Dyslipidemia was defined as participants with at least one of the following criteria: (1) TG ≥2.26 mmol/L (200 mg/dL), (2) TC ≥6.22 mmol/L (240 mg/dL), (3) HDL-C <0.91 mmol/L (35 mg/dL), (4) LDL-C ≥4.14 mmol/L (160 mg/dL), (5) TC-to-HDL-C ratio ≥5, and (6) use of lipid-lowering drug therapy [21]. Anemia was defined as hemoglobin <13 g/dL for men and <12 g/dL for women according to the World Health Organization (WHO) criteria [22].

### 2.3. Dietary Assessment and Other Covariates

Dietary habits were assessed using self-administered standardized and validated semi quantitative food frequency questionnaire (SQ-FFQ) with twenty-two food groups. The frequency and servings of dietary intake were determined at a daily or weekly frequency in the past month. Each food group had five response options (1 to 5) for the frequency and the definition of the portion size (such as a bowl, glass, or serving) as previously described [23]. We also collected other covariates including smoking (none, former, or current), alcohol drinking (yes: ≥1–2 times/week), physical activity (inactive: <1 h/week, active: ≥1–2 h/week), sleep duration (<6 h/day, 6–7 h/day, or >7 h/day), family income (<800,000 NTD, 810,000–1.6 M NTD, or >1.61 M NTD), education (below high school, high school, or above high school), marital status, medical history of CVD, diabetes, or hypertension, and use of cardiovascular, diabetes, or hypertension medication.

### 2.4. Statistical Analysis

**Figure 1:**
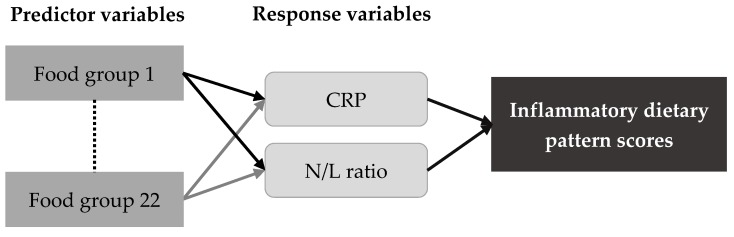
The dietary pattern derived from the reduced rank regression model. CRP: C-reactive protein, N/L: neutrophil-to-lymphocyte.

We used SAS 9.4 (SAS Institute Inc., Cary, NC, USA) and STATA version 13 (StataCorp LP, College Station, TX, USA) for statistical analysis. Continuous and categorical variables were presented as mean ± standard deviation (SD) and number (percentage), respectively. The continuous or categorical variables in the characteristics of study participants were compared between genders using Mann-Whitney U or chi-square test, respectively. Characteristics of the participants across quartiles of dietary pattern scores were analyzed using Kruskal-Wallis test for continuous variables. A linear regression analysis represented by β and 95% confidence intervals (CIs) and a logistic regression analysis represented by odds ratios (ORs) and 95% CIs were performed, respectively, to examine the association of dietary pattern scores with the biomarkers of interest and the risk of dyslipidemia and anemia. The dietary pattern was identified by reduced rank regression (RRR) model using PROC PLS function in SAS 9.4 from 22 food groups as predictor variables and CRP and N/L ratio as response variables (Figure 1). The RRR model is a multivariable linear function, which combines predictor variables (derived from SQ-FFQ) and response variables (refer to nutrients or blood biomarkers) to identify a dietary pattern that was related to disease of interest [24]. The RRR model has been described more detail and documented elsewhere [25,26]. The food groups with the absolute value of factor loading ≥0.20 were used to correspond to the response variables and then derived the dietary pattern associated with inflammation. For further analysis, dietary pattern scores were divided into quartiles and two adjusted models were used: model 1 adjusted for age and BMI and model 2 adjusted for model 1 variables, smoking, alcohol drinking, physical activity, sleep duration, family income, education, marital status, cardiovascular disease, diabetes, hypertension (for dyslipidemia), and dyslipidemia (for anemia). A *p*-value <0.05 was considered statistically significant.

## 3. Results

### 3.1. Characteristics of the Study Participants

Characteristics of the participants between genders are presented in Supplementary Table S1. Males had higher proportion of smoking and alcohol drinking, but lower proportion of physical inactivity than females. The prevalence rates of CVD, diabetes, hypertension, dyslipidemia, and anemia were 6.1%, 9.7%, 28.6%, 32.0%, and 7.9%, respectively. Male participants had higher prevalence of diabetes, hypertension, and dyslipidemia compared with female participants. In contrast, females had higher prevalence of anemia than males (12.9% vs. 3.2%). Correspondingly, females also had lower anemic markers such as hemoglobin (13.1 ± 1.2 g/dL vs. 15.1 ± 1.1 g/dL) and serum iron levels (84.3 ± 33.0 µg/dL vs. 101.9 ± 35.3 µg/dL) than males. Moreover, compared with females, males had higher BMI, blood pressure, inflammatory markers, FBG, TG, LDL-C, TC/HDL-C ratio, BUN, and CRE levels, but lower TC, HDL-C, and eGFR levels (*p* for all < 0.001).

### 3.2. Inflammatory Dietary Pattern and Characteristics Across Quartiles of Dietary Pattern Scores

**Figure 2:**
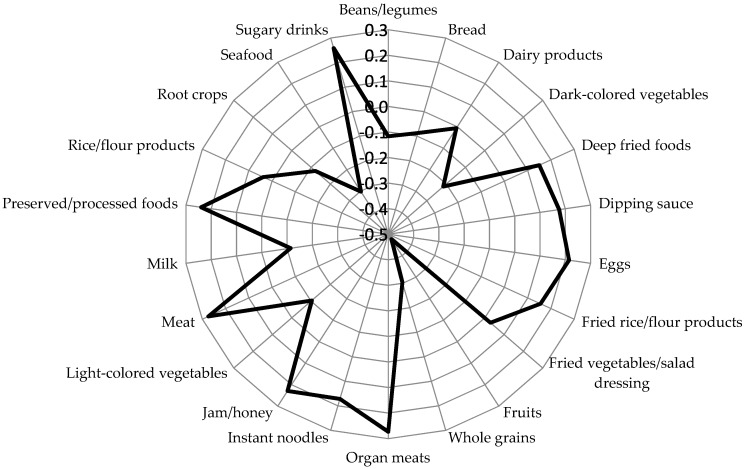
Spider-web diagram of factor loadings of inflammatory dietary pattern identified by reduced rank regression model. Factor loadings are correlations between food groups and dietary pattern scores.

The inflammatory dietary pattern derived by the RRR model showed that meat, organ meats, preserved or processed foods, sugary drinks, jam or honey, and eggs were positively correlated with inflammatory dietary pattern scores (factor loading ≥ 0.20), while fruits, whole grains, seafood, and dark-colored vegetables were negatively associated with inflammatory dietary pattern scores (factor loading ≥ −0.20) (Figure 2). The inflammatory dietary pattern explained 11.6% of total variation. The distribution of quartiles of dietary pattern scores across genders is shown in supplementary Figure S1.

Table 1 shows the characteristics of participants across quartiles of dietary pattern scores. Participants of both genders in the highest quartile (Q4) of inflammatory dietary pattern scores were more likely to be younger, smokers, drinkers, less active, heavier, had better education, and had higher N/L ratio and CRP levels. Male participants in the highest quartile (Q4) of inflammatory dietary pattern scores had higher prevalence of dyslipidemia (42.1%, 38.5%, 37.2%, and 34.5% for Q4, Q3, Q2, and Q1, respectively) and higher TG, TC, LDL-C, and TC/HDL-C ratio, but lower HDL-C levels compared to those in lower quartiles of inflammatory dietary pattern scores. In contrast, females in the highest quartile of inflammatory dietary pattern scores had a lower prevalence of dyslipidemia (23.6% vs. 25.7%) and TC/HDL-C ratio (3.2 ± 0.8 vs. 3.3 ± 0.8), but higher prevalence of anemia (14.7% vs. 11.7%) compared to those in the lowest quartile (Q1) of inflammatory dietary pattern scores. However, there were no significant differences in TG, TC, LDL-C, and HDL-C levels among females in different quartiles of inflammatory dietary pattern scores. In addition, females in the highest quartile of inflammatory dietary pattern scores had lower hemoglobin (13.0 ± 1.2 g/dL vs. 13.2 ± 1.1 g/dL), hematocrit (39.0 ± 3.4% vs. 39.4 ± 3.1%), and serum iron levels (81.7 ± 35.4 µg/dL vs. 84.4 ± 31.0 µg/dL) compared to those in the lowest quartile of inflammatory dietary pattern scores.

### 3.3. Association of Inflammatory Dietary Pattern with Dyslipidemia, Anemia, and Kidney Function Markers

The association between quartiles of inflammatory dietary pattern scores and dyslipidemia is shown in Table 2. After being adjusted by model 2, males in the highest quartile of inflammatory dietary pattern scores had an increased risk of dyslipidemia by 21% (OR = 1.21, 95% CI 1.10–1.34, *p* < 0.001) compared to the reference group (Q1). On the other hand, females with moderate adherence (Q3) of inflammatory dietary pattern scores tended to have an increased risk of dyslipidemia (OR = 1.12, 95% CI 0.99–1.26, *p* = 0.052).

Table 3 demonstrates the association between inflammatory dietary pattern and anemia. The fully adjusted model showed that participants of both genders in higher quartiles of inflammatory dietary pattern scores were associated with an increased risk of anemia by 28% to 47% (Q3: OR = 1.35, 95% CI 1.00–1.80, *p* = 0.045 in males, Q3: OR = 1.28, 95% CI 1.11–1.48, *p* = 0.001 in females, Q4: OR = 1.47, 95% CI 1.26–1.72, *p* < 0.001 in females).

The association of quartiles of inflammatory dietary pattern scores with inflammatory markers, lipid profiles, anemic biomarkers, and kidney function biomarkers is demonstrated in Table 4. The highest quartile of dietary pattern scores were associated with increased CRP, TG, LDL-C, and TC/HDL-C ratio in males and elevated N/L ratio, TG, TC, and LDL-C levels in females (*p* < 0.05). In addition, N/L ratio (Q2, Q3, Q4: 1.84 ± 0.81, 1.85 ± 1.06, 1.89 ± 0.82, *p* trend < 0.001) and TC levels (Q2, Q3, Q4: 5.23 ± 0.88, 5.29 ± 0.92, 5.33 ± 0.90 mmol/L, *p* trend < 0.001) in males were significantly different in all quartiles of inflammatory dietary pattern scores. Females showed a stronger association between quartiles of inflammatory dietary pattern scores and anemic biomarkers compared to males. Females in the highest quartile of inflammatory dietary pattern scores were inversely associated with hemoglobin (β = −0.16, *p* < 0.001), hematocrit (β = −0.38, *p* < 0.001), MCV (β = −1.14, *p* < 0.001), MCH (β = −0.44, *p* < 0.001), MCHC (β = −0.08, *p* < 0.001), and serum iron levels (β = −2.45, *p* < 0.001). While males in the highest quartile of inflammatory dietary pattern scores were correlated with decreased hemoglobin (β = −0.06, *p* = 0.024), MCV (β = −0.35, *p* = 0.017), MCH (β = −0.15, *p* < 0.001), MCHC (β = −0.04, *p* = 0.024), and serum iron levels (β = −2.40, *p* = 0.011). Moreover, participants of both genders in the highest quartile of inflammatory dietary pattern scores (Q4) were associated with increased BUN levels (β = 0.09, *p* = 0.004 in males, β = 0.10, *p* = 0.005 in females) but decreased eGFR levels (β = −0.85, *p* < 0.001 in males, β = −0.53, *p* = 0.022 in females).

## 4. Discussion

To our knowledge, the present study is the first study to identify inflammatory dietary pattern by using the RRR method. Our findings suggest that dietary patterns known to cause inflammation are associated with dyslipidemia in men, anemia in women, and decreased kidney function in both genders. Participants who frequently consumed meat and organ meats were also likely to consume sweetened food or drinks, preserved or processed foods, and eggs. Both meat and organ meats contributed 11.0% to explain the variation in the RRR-derived inflammatory dietary pattern. These food groups were positively associated with inflammatory dietary pattern scores. In contrast, participants who frequently consumed fruits and whole grains were also likely to consume seafood and dark-colored vegetables. Fruits, whole grains, seafood, and dark-colored vegetables contributed 33.4%, 13.6%, 13.4%, and 6.8%, respectively, to explain the variation in the RRR-derived inflammatory dietary pattern. These four food groups comparable as a prudent diet and were negatively associated with inflammatory dietary pattern scores.

The inflammatory dietary pattern identified in the present study was similar to the Western dietary pattern, characterized by high intakes of meat, processed foods, and sweets but low intakes of fruits and vegetables [27,28]. Our study suggests that inflammatory dietary pattern was associated with an increased risk of dyslipidemia by 21% in males, while females consuming inflammatory dietary pattern only showed a borderline increased risk of dyslipidemia (OR = 1.12, 95% CI: 0.99–1.26, *p* = 0.052). Fruits, vegetables, and plant-based food are high in antioxidants and dietary fiber but low in saturated fat, which could have effects on cholesterol metabolism. A previous study reported that a prudent dietary pattern with higher intakes of fruits, vegetables, legumes, seafood, whole grains, and low-fat dairy products significantly decreased blood TC and LDL-C levels [29]. Moreover, for the individual component of blood lipids, high consumption of inflammatory dietary pattern was associated with increased TG, TC, and LDL-C. However, inflammatory dietary pattern was not correlated with HDL-C levels in both genders. Similar to our study, a population-based study in South America also did not find a significant correlation between dietary patterns (both prudent and Western diets) and HDL-C levels in both genders [29]. The authors purposed other factors such as alcohol drinking and physical activity could affect HDL-C levels. However, our study found that there was no association between inflammatory dietary pattern and HDL-C levels despite the increased proportion of alcohol drinkers and physical inactivity across quartiles of inflammatory dietary pattern scores in both genders. Moreover, a meta-analysis of randomized controlled trial also reported that high physical activity combined with a prudent diet was highly effective to reduce TG, TC, and LDL-C levels, but ineffective to increase HDL-C levels [30]. Therefore, a prospective study is necessary to confirm other behavioral effects on blood lipids.

Furthermore, our study found that high consumption of inflammatory dietary pattern was associated with an increased risk of anemia by 28% to 47% and decreased serum iron by at least 2.4 µg/dL in both genders. We also observed that a 1-unit increase in N/L ratio was associated with decreased serum iron levels by 4.89 µg/dL (95% CI, −5.56 to −4.20, *p* < 0.001) in males and by 4.55 µg/dL (95% CI, −5.26 to −3.84, *p* < 0.001) in females (data not shown). There are numerous factors for the development of anemia in participants with declined kidney function. Anemia could be caused by low erythropoietin (EPO) levels. EPO is mainly secreted by the kidney and regulates erythroid proliferation and differentiation in bone marrow. As kidney function declines, the production of EPO is reduced, which leads to decrease the synthesis of red blood cells [31]. Additionally, chronic low-grade inflammation, which was commonly observed in CKD patients, might be also involved in the development of anemia. Elevated hepcidin, stimulated by increased inflammatory cytokines and reduced renal clearance in CKD patients, prevented the use of absorbed iron and/or stored iron for erythropoiesis via the degradation of iron exporter ferroportin and the inhibition of cellular iron efflux [14,32], which consequently resulted in either functional or absolute iron deficiency to meet the demand for erythropoiesis [32]. Other causes for the development of anemia include reduced half-life of circulating blood cells due to uremic toxins and oxidative stress, and folate or vitamin B12 deficiency due to chronic inflammation [31,32].

In the present study, inflammatory dietary pattern was correlated with elevated inflammatory markers (N/L ratio and/or CRP), increased CRE levels and degree of urinary protein (Supplementary Table S2) in men, and decreased eGFR in both genders. Similar to our study, a multi-ethnic cohort study showed that a Western type of dietary pattern rich in meat, processed meat, and sweets had a positive association with CRP levels, interleukin-6, and endothelial dysfunction [12,33,34]. In the present study, we used N/L ratio as inflammatory marker. Recently, high N/L ratio was found to independently predict CVD, degree of proteinuria, and all-cause mortality in CKD patients [16,35]. Additionally, our study also showed that N/L ratio was inversely correlated with eGFR levels (males: β = −0.31, 95% CI: −0.47 to −0.16, *p* < 0.001, females: β = −0.20, 95% CI: −0.37 to −0.02, *p* = 0.028) (Supplementary Table S3). In agreement with the previous studies, elevated inflammatory markers such as CRP were associated with rapid eGFR decline [36,37]. The Western dietary pattern especially consumption of red meat was associated with the reduction of eGFR and the progression to kidney failure in participants with declined kidney function, probably due to a diet with a high acid load [38,39]. In general, foods high in dietary acid load such as processed cheese, meat, and eggs contain exclusively protein and are a source of nonvolatile acids [40]. Nonvolatile acids are produced from the metabolism of organic sulfur or phosphorus-containing amino acids as sulfuric and phosphoric acid [40]. The previous study also showed that a prudent dietary pattern not only ameliorated dyslipidemia but also attenuated oxidized lipoprotein-induced glomerular damage [41]. Higher intakes of plant protein, fruits, and vegetables were associated with higher blood bicarbonate levels, reduced renal acid load, and decreased uremic toxins, which lead to improve kidney filtration function [42,43]. Additionally, a diet rich in fruits, vegetables, and fish had an inverse correlation with plasma CRP levels [33]. Fish intake rich in omega-3 polyunsaturated fatty acids with anti-inflammatory properties was positively associated with kidney CRE clearance rate, indicating that kidney function was improved as increased fish consumption [29,44]. Moreover, inflammatory markers have been considered to be potential mediators for the association between diets and the risk of CVD and mortality [45]. Consistently, our findings suggest that inflammation might be the possible link between dietary patterns and dyslipidemia, anemia, or declined kidney function.

The cause of different results in the association of dietary patterns with the risk of dyslipidemia between genders is not clear yet and may depend on environmental and cultural factors. A previous study in South Africa suggested that the potential factors for the gender differences in the prevalence of disease were food availability and socio-economic status [46]. The motivation to eat healthy might be another factor to influence dietary habits. A previous study in Italy found that women were more willing to follow dietary advice and more aware of the role of nutrition in maintaining health [47]. In our study, men had better income and tended to eat food high in fat, animal protein, and sugary drinks (Supplementary Table S4) as compared to women, and this type of dietary habits in men could potentially increase the risk of developing chronic disease. Additionally, gender-based stereotypes in eating habits could play a potential role. For example, meat rich in fat and protein is food for men, whereas mixed salad with colored vegetables and fruits are food for women [48]. The physiological factors such as sex hormones might affect nutrient metabolism in response to food intake. Although males had higher inflammatory dietary pattern scores than females (0.02 ± 0.93 in males, −0.22 ± 0.87 in females, *p* < 0.001, data not shown), the association of inflammatory dietary pattern with anemia was more prominent in females. The possible reason might be because men consumed heme iron-based food more frequently (Supplementary Table S4). Heme iron is found in meat, poultry, or other animal-based protein. Heme iron is more easily absorbed by enterocytes than non-heme iron, and serves as the predominant source of dietary iron for erythropoiesis [49]. However, prospective and clinical investigation studies are needed to confirm the appropriate intake of heme iron-based food for ameliorating anemia without causing any disease-related symptoms.

Our study has several strengths. First, we used the RRR model to derive the inflammatory dietary pattern. The RRR model could identify diet-and-disease relationship and generate potential mediators between a dietary pattern and a disease. Compared to factor analysis such as principal component analysis, dietary pattern derived from RRR model is more likely to be associated with the disease of interest because the dietary pattern is driven from disease-specific responses [50]. The RRR model allows researchers to extract dietary pattern scores by maximizing explained variation in the response variables or biomarkers which link to diet related disease of interest [51]. However, selecting response variables by the researcher could be personally subjective and may result in different patterns. Second, we used a large sample size from the population with declined kidney function. However, our study has certain limitations. First, the cross-sectional study design cannot identify the causal relationship of the study findings. Prospective observational and clinical intervention studies are necessary to examine the relationship and generate dietary strategies for management of dyslipidemia and anemia in participants with declined kidney function. Secondly, the self-reported questionnaire may have reporting bias. Thirdly, we have adjusted the results with certain confounders, but there are still some potential confounders such as energy and protein intake, which can be considered in a future study. Fourthly, anemia status in this study was only based on hemoglobin levels, and we did not consider iron deficiency anemia because a large number of participants had missing data in transferrin saturation and total iron binding capacity. Measurement of hemoglobin, hematocrit, and iron stores are recommended for clinical diagnosis of anemia in patients with declined kidney function in the future study. Finally, the present study used eGFR levels and urinary protein to define participants with declined kidney function. Hence, we cannot define our participants as CKD patients because CKD cannot be categorized based on a single measurement of eGFR or urinary protein only. Repeated measurements of eGFR or persistent albuminuria for 3 months or more are necessary to confirm clinically diagnosed CKD. Although the MJ Health Institute provided one health examination annually for each member, not all the participants had a periodic health examination to confirm their CKD status. Therefore, our results may not necessarily reflect the association of inflammatory dietary pattern with dyslipidemia, anemia, and kidney function biomarkers among participants with clinically diagnosed CKD.

## 5. Conclusions

The inflammatory dietary pattern may serve as an intermediate factor to understand the relationship between inflammations with the development of chronic diseases. Our findings suggest that participants consuming more inflammatory foods such as meat, organ meats, preserved or processed foods, eggs, sweetened foods or drinks; and lower intake of fruits, whole grains, seafood, and dark-colored vegetables are more likely to have dyslipidemia, anemia, and decreased kidney function.

## Figures and Tables

**Table 1:** Characteristics of the participants across quartiles of inflammatory dietary pattern scores ^a^.

	Dietary Pattern Scores					
	Males			Females		
	Q1 (Low)	Q4 (High)	*p* ^b^	Q1 (Low)	Q4 (High)	*p* ^b^
*n*	4592	6300		5696	3968	
Age (years)	55.3 ± 10.2	48.9 ± 9.0	<0.001	55.4 ± 9.6	49.7 ± 9.0	<0.001
Smoking, current	727 (16.5)	2591 (42.2)	<0.001	63 (1.2)	269 (7.0)	<0.001
Alcohol drinking, yes	1017 (23.8)	1758 (29.5)	<0.001	215 (4.3)	262 (7.2)	<0.001
Physical inactivity	2511 (62.2)	3908 (70.7)	<0.001	3256 (66.9)	2692 (79.0)	<0.001
Family income			<0.001			<0.001
<800,000 NTD	1668 (40.0)	2066 (35.4)		2804 (54.9)	1788 (49.0)	
>1.61 M NTD	853 (20.5)	1263 (21.6)		681 (13.3)	554 (15.2)	
Education			<0.001			<0.001
<high school	889 (19.7)	862 (13.9)		2085 (37.3)	1010 (25.8)	
>high school	1692 (37.5)	2486 (40.0)		1263 (22.6)	1082 (27.6)	
Marital status, married	4031 (93.2)	5308 (88.5)	<0.001	4031 (76.1)	2766 (74.2)	<0.001
Dyslipidemia	1550 (34.5)	2551 (42.1)	<0.001	1449 (25.7)	922 (23.6)	0.04
Anemia	162 (3.5)	154 (2.4)	<0.001	665 (11.7)	584 (14.7)	<0.001
BMI (kg/m^2^)	24.4 ± 2.9	25.0 ± 3.3	<0.001	23.0 ± 3.2	23.3 ± 3.7	<0.001
Inflammatory markers						
N/L ratio	1.8 ± 0.8	1.9 ± 0.9	<0.001	1.8 ± 0.8	1.9 ± 1.0	<0.001
CRP (nmol/L)	21.2 ± 43.8	25.0 ± 46.1	<0.001	20.4 ± 39.9	23.1 ± 53.9	<0.001
Blood lipids						
TG (mmol/L)	1.5 ± 1.0	1.8 ± 1.3	<0.001	1.2 ± 0.7	1.2 ± 0.8	0.08
TC (mmol/L)	5.2 ± 0.9	5.3 ± 0.9	<0.001	5.3 ± 0.9	5.3 ± 0.9	0.32
HDL-C (mmol/L)	1.4 ± 0.3	1.3 ± 0.3	<0.001	1.7 ± 0.4	1.7 ± 0.4	0.16
LDL-C (mmol/L)	3.1 ± 0.8	3.2 ± 0.8	<0.001	3.1 ± 0.8	3.1 ± 0.8	0.22
TC/HDL-C ratio	3.9 ± 0.9	4.1 ± 0.9	<0.001	3.3 ± 0.8	3.2 ± 0.8	0.016
Anemic biomarkers						
RBC (×10^6^/µL)	5.0 ± 0.5	5.1 ± 0.5	<0.001	4.5 ± 0.4	4.5 ± 0.4	<0.001
Hemoglobin (g/dL)	15.0 ± 1.1	15.2 ± 1.1	<0.001	13.2 ± 1.1	13.0 ± 1.2	<0.001
Hematocrit (%)	44.5 ± 3.2	45.2 ± 3.2	<0.001	39.4 ± 3.1	39.0 ± 3.4	<0.001
MCV (fL)	89.8 ± 6.0	89.2 ± 6.3	<0.001	88.5 ± 6.6	87.1 ± 7.5	<0.001
MCH (pg)	30.3 ± 2.4	30.0 ± 2.4	<0.001	29.8 ± 2.6	29.2 ± 3.0	<0.001
MCHC (g/dL)	33.7 ± 0.7	33.6 ± 0.7	<0.001	33.6 ± 0.8	33.5 ± 0.8	<0.001
RDW (%)	13.9 ± 1.1	14.0 ± 1.1	<0.001	13.8 ± 1.3	14.1 ± 1.6	<0.001
Iron (µg/dL)	101.4 ± 34.7	102.4 ± 36.0	0.65	84.8 ± 31.0	81.7 ± 35.4	<0.001
Kidney function biomarkers						
BUN (mmol/L)	5.4 ± 1.4	5.3 ± 1.4	<0.001	5.0 ± 1.5	4.8 ± 1.4	<0.001
CRE (µmol/L)	100.5 ± 16.9	100.6 ± 17.8	0.053	77.4 ± 20.7	76.9 ± 14.2	0.89
eGFR (mL/min/1.73 m^2^)	73.0 ± 9.9	74.5 ± 9.4	<0.001	73.5 ± 10.0	75.1 ± 9.3	<0.001
Proteinuria			0.47			0.54
+1	4375 (95.3)	5952 (94.5)		5514 (96.8)	3828 (96.5)	
+2	119 (2.6)	204 (3.2)		102 (1.8)	82 (2.0)	
≥+3	98 (2.1)	144 (2.3)		80 (1.4)	58 (1.5)	

NTD: new Taiwan dollar, BMI: body mass index, N/L: neutrophil-to-lymphocyte; CRP: C-reactive protein, TG: triglycerides, TC: total cholesterol, HDL-C: high-density lipoprotein cholesterol, LDL-C: low-density lipoprotein cholesterol, TC/HDL-C: total cholesterol-to-high-density lipoprotein cholesterol, RBC: red blood cells, MCV: mean corpuscular volume, MCH: mean corpuscular hemoglobin, MCHC: mean corpuscular hemoglobin concentration, RDW: red blood cell distribution width, BUN: blood urea nitrogen, CRE: creatinine, eGFR: estimated glomerular filtration rate. ^a^ continuous data are presented as mean ± SD and categorical data are presented as number (percentage). ^b^ *p*-value was analyzed using Kruskal-Wallis test for continuous variables and chi-square test for categorical variables.

**Table 2:** Association between quartiles of inflammatory dietary pattern scores and dyslipidemia.

Dietary Pattern Score	Model 1 ^a^		Model 2 ^b^	
	OR (95% CI)	*p*	OR (95% CI)	*p*
Males				
Q1 (low)	Reference			
Q2 (mild)	1.09 (0.99, 1.19)	0.057	1.06 (0.95, 1.17)	0.29
Q3 (moderate)	1.13 (1.04, 1.23)	0.004	1.10 (0.99, 1.21)	0.07
Q4 (high)	1.29 (1.18, 1.40)	<0.001	1.21 (1.10, 1.34)	<0.001
Females				
Q1 (low)	Reference			
Q2 (mild)	1.00 (0.92, 1.10)	0.90	0.94 (0.84, 1.06)	0.32
Q3 (moderate)	1.12 (1.02, 1.23)	0.016	1.12 (0.99, 1.26)	0.052
Q4 (high)	1.10 (0.99, 1.22)	0.06	1.11 (0.98, 1.26)	0.11

^a^ model 1 adjusted for age and BMI. ^b^ model 2 adjusted for model 1 variables, smoking, alcohol drinking, physical activity, sleep duration, family income, education, marital status, cardiovascular disease, diabetes, and hypertension.

**Table 3:** Association between quartiles of inflammatory dietary pattern scores and anemia.

Dietary Pattern Score	Model 1 ^a^		Model 2 ^b^	
	OR (95% CI)	*p*	OR (95% CI)	*p*
Males				
Q1 (low)	Reference			
Q2 (mild)	1.29 (1.04, 1.61)	0.021	1.29 (0.97, 1.69)	0.07
Q3 (moderate)	1.26 (1.01, 1.57)	0.044	1.35 (1.00, 1.80)	0.045
Q4 (high)	1.15 (0.91, 1.44)	0.25	1.24 (0.93, 1.67)	0.14
Females				
Q1 (low)	Reference			
Q2 (mild)	1.06 (0.94, 1.91)	0.32	1.11 (0.96, 1.27)	0.16
Q3 (moderate)	1.19 (1.06, 1.34)	0.003	1.28 (1.11, 1.48)	0.001
Q4 (high)	1.30 (1.15, 1.47)	<0.001	1.47 (1.26, 1.72)	<0.001

^a^ model 1 adjusted for age and BMI. ^b^ model 2 adjusted for model 1 variables, smoking, alcohol drinking, physical activity, sleep duration, family income, education, marital status, cardiovascular disease, diabetes, hypertension, and dyslipidemia.

**Table 4:** Linear association of quartiles of inflammatory dietary pattern scores with inflammatory markers, lipid profiles, anemic biomarkers, and kidney function biomarkers ^a^.

	Dietary Pattern Score						
	Q1 (Ref)	Q2 (Mild)		Q3 (Moderate)		Q4 (High)	
		β (95% CI)	*p*	β (95% CI)	*p*	β (95% CI)	*p*
Males							
Inflammatory markers							
N/L ratio	0	0.05 (0.01, 0.09)	0.02	0.06 (0.02, 0.10)	0.005	0.12 (0.08, 0.16)	<0.001
CRP (nmol/L)	0	1.75 (−0.70, 4.20)	0.16	2.68 (0.25, 5.12)	0.031	3.82 (1.38, 6.26)	0.002
Blood lipids							
TG (mmol/L)	0	0.03 (−0.02, 0.08)	0.28	0.04 (−0.01, 0.09)	0.13	0.09 (0.04, 0.14)	0.001
TC (mmol/L)	0	0.05 (0.01, 0.09)	0.013	0.10 (0.06, 0.14)	<0.001	0.12 (0.08, 0.16)	<0.001
HDL-C (mmol/L)	0	−0.00 (−0.02, 0.01)	0.72	0.00 (−0.01, 0.01)	0.92	−0.01 (−0.02, 0.00)	0.13
LDL-C (mmol/L)	0	0.04 (−0.00, 0.07)	0.07	0.08 (0.04, 0.12)	<0.001	0.09 (0.05, 0.12)	<0.001
TC/HDL-C ratio	0	0.04 (−0.01, 0.08)	0.09	0.06 (0.02, 0.11)	0.002	0.11 (0.07, 0.15)	<0.001
Anemic biomarkers ^b^							
RBC (×10^6^/µL)	0	0.01 (−0.02, 0.03)	0.53	0.01 (−0.01, 0.04)	0.23	0.01 (−0.01, 0.03)	0.55
Hemoglobin (g/dL)	0	−0.02 (−0.07, 0.03)	0.41	−0.03 (−0.08, 0.02)	0.27	−0.06 (−0.11, −0.01)	0.024
Hematocrit (%)	0	−0.05 (−0.19, 0.10)	0.50	−0.05 (−0.19, 0.10)	0.50	−0.12 (−0.26, 0.02)	0.11
MCV (fL)	0	−0.22 (−0.51, 0.07)	0.14	−0.31 (−0.60, −0.02)	0.034	−0.35 (−0.63, −0.06)	0.017
MCH (pg)	0	−0.08 (−0.20, 0.03)	0.14	−0.13 (−0.24, −0.01)	0.028	−0.15 (−0.26, −0.04)	<0.001
MCHC (g/dL)	0	−0.01 (−0.04, 0.02)	0.54	−0.02 (−0.06, 0.01)	0.16	−0.04 (−0.07, −0.01)	0.024
RDW (%)	0	0.03 (−0.02, 0.08)	0.31	0.08 (0.03, 0.13)	0.003	0.08 (0.03, 0.13)	0.003
Iron (µg/dL)	0	−2.22 (−4.07, −0.36)	0.019	−1.71 (−3.56, 0.14)	0.07	−2.40 (−4.25, −0.54)	0.011
Kidney function biomarkers							
BUN (mmol/L)	0	0.11 (0.04, 0.17)	0.001	0.09 (0.03, 0.15)	0.004	0.09 (0.03, 0.15)	0.004
CRE (µmol/L)	0	1.12 (0.22, 2.03)	0.015	1.41 (0.51, 2.30)	0.002	1.51 (0.62, 2.40)	0.001
eGFR (mL/min/1.73 m^2^)	0	−0.52 (−0.94, −0.10)	0.015	−0.62 (−1.03, −0.20)	0.004	−0.85 (−1.26, −0.43)	<0.001
Females							
Inflammatory markers							
N/L ratio	0	0.02 (−0.02, 0.06)	0.36	0.07 (0.03, 0.11)	0.001	0.13 (0.09, 0.17)	<0.001
CRP (nmol/L)	0	0.63 (−1.45, 2.71)	0.55	0.92 (−1.23, 3.08)	0.40	2.30 (−0.03, 4.62)	0.053
Blood lipids							
TG (mmol/L)	0	0.02 (−0.02, 0.05)	0.31	0.03 (−0.00, 0.06)	0.09	0.06 (0.02, 0.09)	0.001
TC (mmol/L)	0	0.02 (−0.02, 0.07)	0.21	0.06 (0.02, 0.11)	0.002	0.08 (0.04, 0.13)	<0.001
HDL-C (mmol/L)	0	−0.00 (−0.02, 0.02)	0.80	−0.00 (−0.02, 0.02)	0.74	0.01 (−0.01, 0.03)	0.44
LDL-C (mmol/L)	0	0.02 (−0.01, 0.06)	0.19	0.06 (0.02, 0.09)	0.003	0.05 (0.01, 0.09)	0.019
TC/HDL-C ratio	0	0.02 (−0.02, 0.05)	0.34	0.05 (0.02, 0.09)	0.005	0.04 (−0.00, 0.08)	0.06
Anemic biomarkers ^b^							
RBC (×10^6^/µL)	0	−0.01 (−0.03, 0.01)	0.21	−0.01 (−0.03, 0.01)	0.19	0.02 (−0.00, 0.04)	0.13
Hemoglobin (g/dL)	0	−0.05 (−0.10, 0.00)	0.07	−0.09 (−0.14, −0.03)	0.002	−0.16 (−0.22, −0.10)	<0.001
Hematocrit (%)	0	−0.15 (−0.29, −0.01)	0.041	−0.19 (−0.34, −0.04)	0.012	−0.38 (−0.54, −0.22)	<0.001
MCV (fL)	0	−0.14 (−0.46, 0.17)	0.37	−0.22 (−0.55, 0.11)	0.19	−1.14 (−1.49, −0.79)	<0.001
MCH (pg)	0	−0.04 (−0.17, 0.09)	0.53	−0.05 (−0.19, 0.08)	0.41	−0.44 (−0.58, −0.30)	<0.001
MCHC (g/dL)	0	0.01 (−0.03, 0.04)	0.71	0.02 (−0.02, 0.06)	0.28	−0.08 (−0.12, −0.04)	<0.001
RDW (%)	0	0.05 (−0.02, 0.12)	0.16	0.04 (−0.03, 0.11)	0.24	0.15 (0.08, 0.23)	<0.001
Iron (µg/dL)	0	0.50 (−1.17, 2.14)	0.58	0.95 (−0.77, 2.67)	1.08	−2.45 (−4.31, −0.60)	0.01
Kidney function biomarkers							
BUN (mmol/L)	0	0.04 (−0.02, 0.10)	0.18	0.16 (0.09, 0.22)	<0.001	0.10 (0.03, 0.16)	0.005
CRE (µmol/L)	0	−0.12 (−0.91, 0.67)	0.77	1.05 (0.23, 1.86)	0.012	0.61 (−0.26, 1.48)	0.17
eGFR (mL/min/1.73 m^2^)	0	−0.03 (−0.44, 0.38)	0.89	−0.54 (−0.96, −0.11)	0.013	−0.53 (−0.98, −0.08)	0.022

N/L: neutrophil-to-lymphocyte; CRP: C-reactive protein, TG: triglycerides, TC: total cholesterol, HDL-C: high-density lipoprotein cholesterol, LDL-C: low-density lipoprotein cholesterol, TC/HDL-C: total cholesterol-to-high-density lipoprotein cholesterol, RBC: red blood cells, MCV: mean corpuscular volume, MCH: mean corpuscular hemoglobin, MCHC: mean corpuscular hemoglobin concentration, RDW: red blood cell distribution width, BUN: blood urea nitrogen, CRE: creatinine, eGFR: estimated glomerular filtration rate. Quartile 1 of inflammatory dietary pattern scores were used as reference. ^a^ adjusted by model 2: age, BMI, smoking, alcohol drinking, physical activity, sleep duration, family income, education, marital status, cardiovascular disease, diabetes, and hypertension. ^b^ adjusted for model 2 variables and dyslipidemia.

## Supplementary Materials

The followings are available online at https://www.mdpi.com/2072-6643/11/9/2052/s1, Table S1: Characteristics of the study participants across genders, Table S2: Multivariate logistic regression analysis of dietary pattern scores across quartiles with proteinuria severity, Table S3: Multivariate linear regression analysis of N/L ratio with kidney function biomarkers, Table S4: Intake frequency of the food groups by different genders. Figure S1: Distribution of dietary pattern scores among males and females.

## References

[1] Saland J.M., Ginsberg H.N. (2007). Lipoprotein metabolism in chronic renal insufficiency. Pediatr. Nephrol..

[2] Lee P.H., Chang H.Y., Tung C.W., Hsu Y.C., Lei C.C., Chang H.H., Yang H.F., Lu L.C., Jong M.C., Chen C.Y. (2009). Hypertriglyceridemia: An independent risk factor of chronic kidney disease in taiwanese adults. Am. J. Med. Sci..

[3] Baragetti A., Norata G.D., Sarcina C., Rastelli F., Grigore L., Garlaschelli K., Uboldi P., Baragetti I., Pozzi C., Catapano A.L. (2013). High density lipoprotein cholesterol levels are an independent predictor of the progression of chronic kidney disease. J. Intern. Med..

[4] Wang Y.N., Qiu X.L., Lv L.S., Wang C.X., Ye Z.C., Li S.M., Liu Q., Lou T., Liu X. (2016). Correlation between serum lipid levels and measured glomerular filtration rate in chinese patients with chronic kidney disease. PLoS ONE.

[5] Chen S.C., Hung C.C., Kuo M.C., Lee J.J., Chiu Y.W., Chang J.M., Hwang S.J., Chen H.C. (2013). Association of dyslipidemia with renal outcomes in chronic kidney disease. PLoS ONE.

[6] Inker L.A., Coresh J., Levey A.S., Tonelli M., Muntner P. (2011). Estimated GFR, albuminuria, and complications of chronic kidney disease. J. Am. Soc. Nephrol..

[7] Stauffer M.E., Fan T. (2014). Prevalence of anemia in chronic kidney disease in the United States. PLoS ONE.

[8] Levin A., Thompson C.R., Ethier J., Carlisle E.J., Tobe S., Mendelssohn D., Burgess E., Jindal K., Barrett B., Singer J. (1999). Left ventricular mass index increase in early renal disease: Impact of decline in hemoglobin. Am. J. Kidney Dis..

[9] Astor B.C., Coresh J., Heiss G., Pettitt D., Sarnak M.J. (2006). Kidney function and anemia as risk factors for coronary heart disease and mortality: The Atherosclerosis Risk in Communities (ARIC) Study. Am. Heart J..

[10] Chang J.M., Chen S.C., Huang J.C., Su H.M., Chen H.C. (2014). Anemia and left ventricular hypertrophy with renal function decline and cardiovascular events in chronic kidney disease. Am. J. Med. Sci..

[11] Gupta J., Mitra N., Kanetsky P.A., Devaney J., Wing M.R., Reilly M., Shah V.O., Balakrishnan V.S., Guzman N.J., Girndt M. (2012). Association between albuminuria, kidney function, and inflammatory biomarker profile in CKD in CRIC. Clin. J. Am. Soc. Nephrol..

[12] Barbaresko J., Koch M., Schulze M.B., Nothlings U. (2013). Dietary pattern analysis and biomarkers of low-grade inflammation: A systematic literature review. Nutr. Rev..

[13] Stenvinkel P., Heimbürger O., Paultre F., Diczfalusy U., Wang T., Berglund L., Jogestrand T. (1999). Strong association between malnutrition, inflammation, and atherosclerosis in chronic renal failure. Kidney Int..

[14] Kutuby F., Wang S., Desai C., Lerma E.V. (2015). Anemia of chronic kidney disease. Dis. Mon..

[15] Okyay G.U., Inal S., Onec K., Er R.E., Paşaoğlu O., Paşaoğlu H., Derici U., Erten Y. (2013). Neutrophil to lymphocyte ratio in evaluation of inflammation in patients with chronic kidney disease. Ren. Fail..

[16] Lu X.X., Wang S.X., Zhang G.Z., Xiong R.F., Li H. (2018). High neutrophil-to-lymphocyte ratio is a significant predictor of cardiovascular and all-cause mortality in patients undergoing peritoneal dialysis. Kidney Blood Press. Res..

[17] Knight E.L., Stampfer M.J., Hankinson S.E., Spiegelman D., Curhan G.C. (2003). The impact of protein intake on renal function decline in women with normal renal function or mild renal insufficiency. Ann. Intern. Med..

[18] de Oliveira Otto M.C., Mozaffarian D., Kromhout D., Bertoni A.G., Sibley C.T., Jacobs D.R., Nettleton J.A. (2012). Dietary intake of saturated fat by food source and incident cardiovascular disease: The Multi-Ethnic Study of Atherosclerosis. Am. J. Clin. Nutr..

[19] Hu F.B. (2002). Dietary pattern analysis: A new direction in nutritional epidemiology. Curr. Opin. Lipidol..

[20] Schwandt A., Denkinger M., Fasching P., Pfeifer M., Wagner C., Weiland J., Zeyfang A., Holl R.W. (2017). Comparison of MDRD, CKD-EPI, and Cockcroft-Gault equation in relation to measured glomerular filtration rate among a large cohort with diabetes. J. Diabetes Complicat..

[21] Chang H.Y., Yeh W.T., Chang Y.H., Tsai K.S., Pan W.H. (2002). Prevalence of dyslipidemia and mean blood lipid values in Taiwan: Results from the Nutrition and Health Survey in Taiwan (NAHSIT, 1993-1996). Chin. J. Physiol..

[22] World Health Organization (2001). Iron Deficiency Anemia: Assessment, Prevention and Control. A Guide for Programme Managers.

[23] Muga M.A., Owili P.O., Hsu C.Y., Rau H.H., Chao J.C.J. (2016). Association between dietary patterns and cardiovascular risk factors among middle-aged and elderly adults in Taiwan: A population-based study from 2003 to 2012. PLoS ONE.

[24] Kurniawan A.L., Hsu C.Y., Rau H.H., Lin L.Y., Chao J.C. (2019). Association of kidney function-related dietary pattern, weight status, and cardiovascular risk factors with severity of impaired kidney function in middle-aged and older adults with chronic kidney disease: A cross-sectional population study. Nutr. J..

[25] Hoffmann K., Schulze M.B., Schienkiewitz A., Nothlings U., Boeing H. (2004). Application of a new statistical method to derive dietary patterns in nutritional epidemiology. Am. J. Epidemiol..

[26] Weikert C., Schulze M.B. (2016). Evaluating dietary patterns: The role of reduced rank regression. Curr. Opin. Clin. Nutr. Metab. Care.

[27] Paterson E.N., Neville C.E., Silvestri G., Montgomery S., Moore E., Silvestri V., Cardwell C.R., MacGillivray T.J., Maxwell A.P., Woodside J.V. (2018). Dietary patterns and chronic kidney disease: A cross-sectional association in the Irish Nun Eye Study. Sci. Rep..

[28] Lin J.L., Fung T.T., Hu F.B., Curhan G.C. (2011). Association of dietary patterns with albuminuria and kidney function decline in older white women: A subgroup analysis from the Nurses’ Health Study. Am. J. Kidney Dis..

[29] Poggio R., Elorriaga N., Gutierrez L., Irazola V., Rubinstein A., Danaei G. (2017). Associations between dietary patterns and serum lipids, apo and C-reactive protein in an adult population: Evidence from a multi-city cohort in South America. Br. J. Nutr..

[30] Kelley G.A., Kelley K.S., Roberts S., Haskell W. (2011). Efficacy of aerobic exercise and a prudent diet for improving selected lipids and lipoproteins in adults: A meta-analysis of randomized controlled trials. BMC Med..

[31] Atkinson M.A., Warady B.A. (2017). Anemia in chronic kidney disease. Pediatr. Nephrol..

[32] Babitt J.L., Lin H.Y. (2012). Mechanisms of anemia in CKD. J. Am. Soc. Nephrol..

[33] Lopez-Garcia E., Schulze M.B., Fung T.T., Meigs J.B., Rifai N., Manson J.E., Hu F.B. (2004). Major dietary patterns are related to plasma concentrations of markers of inflammation and endothelial dysfunction. Am. J. Clin. Nutr..

[34] Nettleton J.A., Steffen L.M., Mayer-Davis E.J., Jenny N.S., Jiang R., Herrington D.M., Jacobs D.R. (2006). Dietary patterns are associated with biochemical markers of inflammation and endothelial activation in the Multi-Ethnic Study of Atherosclerosis (MESA). Am. J. Clin. Nutr..

[35] Binnetoğlu E., Şengül E., Halhallı G., Dindar S., Şen H. (2014). Is neutrophil lymphocyte ratio an indicator for proteinuria in chronic kidney disease?. J. Clin. Lab. Anal..

[36] Amdur R.L., Feldman H.I., Gupta J., Yang W., Kanetsky P., Shlipak M., Rahman M., Lash J.P., Townsend R.R., Ojo A. (2016). Inflammation and progression of CKD: The CRIC study. Clin. J. Am. Soc. Nephrol..

[37] Tonelli M., Sacks F., Pfeffer M., Jhangri G.S., Curhan G. (2005). Cholesterol and Recurrent Events (CARE) Trial Investigators. Biomarkers of inflammation and progression of chronic kidney disease. Kidney Int..

[38] Lew Q.J., Jafar T.H., Koh H.W., Jin A., Chow K.Y., Yuan J.M., Koh W.P. (2017). Red meat intake and risk of ESRD. J. Am. Soc. Nephrol..

[39] Banerjee T., Crews D.C., Wesson D.E., Tilea A.M., Saran R., Rios-Burrows N., Williams D.E., Powe N.R., Centers for Disease Control and Prevention Chronic Kidney Disease Surveillance Team (2015). High dietary acid load predicts ESRD among adults with CKD. J. Am. Soc. Nephrol..

[40] Scialla J.J., Anderson C.A. (2013). Dietary acid load: A novel nutritional target in chronic kidney disease?. Adv. Chronic Kidney Dis..

[41] Chen X., Wei G., Jalili T., Metos J., Giri A., Cho M.E., Boucher R., Greene T., Beddhu S. (2016). The associations of plant protein intake with all-cause mortality in CKD. Am. J. Kidney Dis..

[42] Goraya N., Simoni J., Jo C.H., Wesson D.E. (2013). A comparison of treating metabolic acidosis in CKD stage 4 hypertensive kidney disease with fruits and vegetables or sodium bicarbonate. Clin. J. Am. Soc. Nephrol..

[43] Scialla J.J., Appel L.J., Wolf M., Yang W., Zhang X.M., Sozio S.M., Miller E.R., Bazzano L.A., Cuevas M., Glenn M.J. (2012). Plant protein intake is associated with fibroblast growth factor 23 and serum bicarbonate levels in patients with chronic kidney disease: The Chronic Renal Insufficiency Cohort study. J. Ren. Nutr..

[44] Chrysohoou C., Pitsavos C., Panagiotakos D., Skoumas J., Lazaros G., Oikonomou E., Galiatsatos N., Striggou M., Xynogala M., Stefanadis C. (2013). Long-term fish intake preserves kidney function in elderly individuals: The Ikaria study. J. Ren. Nutr..

[45] Zhong X., Guo L., Zhang L., Li Y., He R., Cheng G. (2017). Inflammatory potential of diet and risk of cardiovascular disease or mortality: A meta-analysis. Sci. Rep..

[46] Case A., Menendez A. (2009). Sex differences in obesity rates in poor countries: Evidence from South Africa. Econ. Hum. Biol..

[47] Leblanc V., Begin C., Corneau L., Dodin S., Lemieux S. (2015). Gender differences in dietary intakes: What is the contribution of motivational variables?. J. Hum. Nutr. Diet..

[48] Masella R., Malorni W. (2017). Gender-related differences in dietary habits. Clin. Manag. Issues.

[49] Pizarro F., Olivares M., Hertrampf E., Mazariegos D.I., Arredondo M. (2003). Heme-iron absorbtion is saturable by heme-iron dose in women. J. Nutr..

[50] Manios Y., Kourlaba G., Grammatikaki E., Androutsos O., Ioannou E., Roma-Giannikou E. (2010). Comparison of two methods for identifying dietary patterns associated with obesity in preschool children: The GENESIS study. Eur. J. Clin. Nutr..

[51] Heidemann C., Hoffmann K., Spranger J., Klipstein-Grobusch K., Möhlig M., Pfeiffer A., Boeing H., European Prospective Investigation into Cancer and Nutrition (EPIC)-Potsdam Study Cohort (2005). A dietary pattern protective against type 2 diabetes in the European Prospective Investigation into Cancer and Nutrition (EPIC)-Potsdam Study cohort. Diabetologia.

