# Supplementary material for: Inflammatory Dietary Pattern Predicts Dyslipidemia and Anemia in Middle-Aged and Older Taiwanese Adults with Declined Kidney Function: A Cross-Sectional Population Study from 2008 to 2010
Source: Nutrients. 2019 Sep 2;11(9):2052. doi: 10.3390/nu11092052 (PMC6770658; doi:10.3390/nu11092052)
Supplement: Supplementary file 1 [file nutrients-11-02052-s001.zip › nutrients-549723-SI.pdf]

Inflammatory Dietary Pattern Predicts Dyslipidemia and Anemia in Middle-aged and Older Taiwanese Adults with Declined Kidney Function: A Cross-Sectional Population Study from 2008 to 2010

Adi Lukas Kurniawan [1], Chien-Yeh Hsu [2,3], Hsiao-Hsien Rau [4], Li-Yin Lin[1], and Jane C-J Chao [1,3,5*]

Table S1. Characteristics of the study participants across genders (n = 41,128) [a].

	**Total** (***n*** **= 41,128**)	**Males** (n = 21,376)	**Females** (n = 19,752)	p [b]
Age (years)	52.6 ± 9.9	51.9 ± 0.1	53.3 ± 0.1	<0.001
Smoking, current	6717 (17.0)	6086 (28.5)	631 (3.2)	<0.001
Alcohol drinking, yes	6420 (17.0)	5405 (25.3)	1015 (5.1)	<0.001
Physical inactivity	24726 (60.1)	12554 (58.7)	12172 (61.6)	<0.001
Sleep duration				<0.001
<6 hours/day	10575 (25.7)	4828 (22.6)	5747 (29.1)	
>7 hours/day	9707 (23.6)	5325 (24.9)	4382 (22.2)	
Family income				<0.001
<800,000 NTD	16891 (41.1)	7395 (34.6)	9496 (48.1)	
>1.61 M NTD	6481 (15.8)	4036 (18.9)	2445 (12.4)	
Education				<0.001
< high school	10215 (24.8)	3583 (16.8)	6632 (33.6)	
> high school	12697 (30.9)	8150 (38.1)	4547 (23.0)	
Marital status, married	32286 (78.5)	18412 (86.1)	13874 (70.2)	<0.001
Cardiovascular disease [c]	2489 (6.1)	1298 (6.1)	1191 (6.0)	0.86
Diabetes [d]	3974 (9.7)	2356 (11.0)	1618 (8.2)	<0.001
Hypertension [e]	11779 (28.6)	6762 (31.6)	5017 (25.4)	<0.001
Dyslipidemia	12909 (32.0)	7967 (37.3)	4942 (25.0)	<0.001
Anemia	3232 (7.9)	684 (3.2)	2548 (12.9)	<0.001
BMI (kg/m^2)	24.0 ± 3.3	24.7 ± 3.1	23.2 ± 3.4	<0.001
SBP (mmHg)	122.5 ± 18.2	124.7 ± 16.7	120.1 ± 19.4	<0.001
DBP (mmHg)	73.8 ± 11.8	76.9 ± 11.2	70.6 ± 11.5	<0.001
Inflammatory markers				
Leukocytes (×10^3/μL)	5.9 ± 1.7	6.1 ± 1.6	5.6 ± 1.7	<0.001
Neutrophils (×10^3/μL)	3.3 ± 1.3	3.4 ± 1.2	3.2 ± 1.4	<0.001
Lymphocytes (×10^3/μL)	1.9 ± 0.6	2.0 ± 0.6	1.8 ± 0.6	<0.001
N/L ratio	1.8 ± 0.9	1.9 ± 0.9	1.8 ± 0.9	<0.001
CRP (nmol/L)	22.5 ± 47.9	22.8 ± 46.9	21.6 ± 47.9	<0.001

Blood sugar and lipids				
FBG (mmol/L)	5.9 ± 1.3	6.0 ± 1.3	5.8 ± 1.3	<0.001
TG (mmol/L)	1.4 ± 1.1	1.5 ± 0.8	1.2 ± 0.7	<0.001
TC (mmol/L)	5.3 ± 0.9	5.2 ± 0.9	5.3 ± 0.9	<0.001
HDL-C (mmol/L)	1.5 ± 0.4	1.4 ± 0.3	1.7 ± 0.4	<0.001
LDL-C (mmol/L)	3.1 ± 0.8	3.2 ± 0.8	3.1 ± 0.8	<0.001
TC/HDL-C ratio	3.6 ± 0.9	4.0 ± 0.9	3.3 ± 0.8	<0.001
Anemic biomarkers				
RBC ($\times 10^6$/μL)	4.7 ± 0.5	5.0 ± 0.5	4.5 ± 0.4	<0.001
Hemoglobin (g/dL)	14.2 ± 1.5	15.1 ± 1.1	13.1 ± 1.2	<0.001
Hematocrit (%)	42.2 ± 4.3	44.9 ± 3.2	39.2 ± 3.2	<0.001
MCV (fL)	88.7 ± 6.6	89.4 ± 6.2	88.0 ± 7.0	<0.001
MCH (pg)	29.9 ± 2.6	30.1 ± 2.5	29.6 ± 2.8	<0.001
MCHC (g/dL)	33.6 ± 0.8	33.7 ± 0.7	33.6 ± 0.8	<0.001
RDW (%)	13.9 ± 1.3	14.0 ± 1.1	13.9 ± 1.5	<0.001
Iron (μg/dL)	93.1 ± 35.3	101.9 ± 35.3	84.3 ± 33.0	<0.001
Kidney function biomarkers				
BUN (mmol/L)	5.2 ± 1.5	5.4 ± 1.5	5.0 ± 1.5	<0.001
CRE (μmol/L)	89.5 ± 22.2	101.0 ± 20.8	77.4 ± 18.7	<0.001
eGFR (mL/min/1.73 m^2)	73.7 ± 9.9	73.7 ± 9.8	74.1 ± 9.9	<0.001
Proteinuria				<0.001
+1	39311 (95.6)	20260 (94.8)	19051 (96.5)	
+2	1041 (2.5)	641 (3.0)	400 (2.0)	
≥+3	776 (1.9)	475 (2.2)	301 (1.5)	

NTD: new Taiwan dollar, BMI: body mass index, SBP: systolic blood pressure, DBP: diastolic blood pressure, N/L, neutrophil-to-lymphocyte; CRP: C-reactive protein, FBG: fasting blood glucose, TG: triglycerides, TC: total cholesterol, HDL-C: high-density lipoprotein cholesterol, LDL-C: low-density lipoprotein cholesterol, TC/HDL-C: total cholesterol-to-high-density lipoprotein cholesterol, RBC: red blood cells, MCV: mean corpuscular volume, MCH: mean corpuscular hemoglobin, MCHC: mean corpuscular hemoglobin concentration, RDW: red blood cell distribution width, BUN: blood urea nitrogen, CRE: creatinine, eGFR: estimated glomerular filtration rate. [a] continuous data are presented as mean ± SD and categorical data are presented as number (percentage). [b] *p*-values were analyzed using Mann-Whitney U test for continuous variables and chi-square test for categorical variables. [c] cardiovascular disease: having a history of cardiovascular disease or use of cardiovascular drug. [d] diabetes: having a history of diabetes, use of hypoglycemic drug or FBG ≥7.0 mmol/L (126 mg/dL). [e] hypertension: having a history of hypertension, use of hypertension drug, systolic pressure ≥140 mmHg or diastolic pressure ≥90 mmHg.

Table S2. Multivariate logistic regression analysis of dietary pattern scores across quartiles with proteinuria severity.

**Dietary Pattern Scores**	**Model 1** [a]		**Model 2** [b]	
	**OR (95% CI)**	***p***	**OR (95%CI)**	***p***
Males				

Q1 (ref)	1		1	
Q2 (mild)	1.26 (1.04, 1.51)	0.02	1.23 (0.97, 1.56)	0.09
Q3 (moderate)	1.20 (1.00, 1.45)	0.05	1.27 (1.01, 161)	0.044
Q4 (high)	1.47 (1.23, 1.76)	<0.001	1.44 (1.14, 1.83)	0.002
Females				
Q1 (ref)	1		1	
Q2 (mild)	1.12 (0.91, 1.38)	0.29	1.06 (0.81, 1.39)	0.69
Q3 (moderate)	1.32 (1.07, 1.63)	0.009	1.30 (0.99, 1.71)	0.06
Q4 (high)	1.31 (1.04, 1.65)	0.02	1.30 (0.97, 1.75)	0.08

[a] model 1 adjusted for age and BMI. [b] model 2 adjusted for model 1 variables, smoking, alcohol drinking, physical activity, sleep duration, family income, education, marital status, cardiovascular disease, diabetes, and hypertension. Proteinuria severity was analyzed as a dummy variable with +1 served as a reference.

Table S3. Multivariate linear regression analysis of N/L ratio with kidney function biomarkers.

Dependent Variables	Model 1 [a]		Model 2 [b]	
	β (95% CI)	*p*	β (95%CI)	*p*
		Males		
CRP (nmol/L)	10.17 (9.40, 10.93)	<0.001	9.98 (9.08, 10.88)	<0.001
BUN (mmol/L)	0.05 (0.03, 0.07)	<0.001	0.04 (0.01, 0.06)	0.003
CRE (μmol/L)	1.11 (0.81, 1.41)	<0.001	0.96 (0.62, 1.30)	<0.001
eGFR (mL/min/1.73 m^2)	-0.36 (-0.50, -0.22)	<0.001	-0.31 (-0.47, -0.16)	<0.001
		Females		
CRP (nmol/L)	13.14 (12.35, 13.94)	<0.001	12.20 (11.33, 13.08)	<0.001
BUN (mmol/L)	0.01 (-0.02, 0.03)	0.506	0.00 (-0.02, 0.03)	0.816
CRE (μmol/L)	1.14 (0.85, 1.43)	<0.001	1.03 (0.70, 1.37)	<0.001
eGFR (mL/min/1.73 m^2)	-0.30 (-0.45, -0.16)	<0.001	-0.20 (-0.37, -0.02)	0.028

CRP: C-reactive protein, BUN: blood urea nitrogen, CRE: creatinine, eGFR: estimated glomerular filtration rate. [a] model 1 adjusted for age and BMI. [b] model 2 adjusted for model 1 variables, smoking, alcohol drinking, physical activity, sleep duration, family income, education, marital status, cardiovascular disease, diabetes, and hypertension.

Table S4. Intake frequency of the food groups by different genders (n = 41,128) [a].

Food Groups	Males (n = 21,376)	Females (n = 19,752)	p
Bread [b]	1601 (7.5)	1605 (8.1)	0.017
Whole grains [b]	3323 (15.5)	2961 (15.0)	0.12
Beans/legumes [b]	1901 (8.9)	1888 (9.6)	0.02
Seafood [b]	2192 (10.3)	1862 (9.4)	0.005
Eggs [b]	2098 (9.8)	987 (5.0)	<0.001
Meat [b]	5724 (26.8)	3644 (18.4)	<0.001
Organ meats [b]	86 (0.4)	44 (0.2)	0.002
Dairy products [b]	476 (2.2)	612 (3.1)	<0.001
Deep fried foods [b]	677 (3.2)	590 (3.0)	0.30
Fried rice/flour products [b]	733 (3.4)	690 (3.5)	0.73
Instant noodles [b]	30 (0.1)	14 (0.1)	0.035
Preserved/processed foods [b]	155 (0.7)	118 (0.6)	0.12
Dipping sauce [b]	616 (2.9)	417 (2.1)	<0.001
Jam/honey [b]	602 (2.8)	524 (2.7)	0.32
Rice/flour products [c]	1393 (6.5)	392 (2.0)	<0.001
Fruits [c]	535 (2.5)	571 (2.9)	<0.001
Root crops [d]	453 (2.1)	561 (2.8)	<0.001
Dark-colored vegetables [e]	1207 (5.6)	1548 (7.8)	<0.001
Light-colored vegetables [e]	1519 (7.1)	1651 (8.4)	0.016
Fried vegetables/salad dressing [e]	494 (2.3)	638 (3.2)	<0.001
Milk [f]	2317 (10.8)	2574 (13.0)	<0.001
Sugary drinks [f]	3535 (16.5)	2705 (13.7)	<0.001

[a] Data are expressed as number (percentage). The number of the participants was shown for those who consumed the food group at least the 4th high intake frequency in 5 different frequency options in the food frequency questionnaire. [b] consumed ≥1 serving/day. [c] consumed ≥3 servings/day. [d] consumed ≥½ bowl/day. [e] consumed ≥1½ bowls/day. [f] consumed ≥1 cup/day.

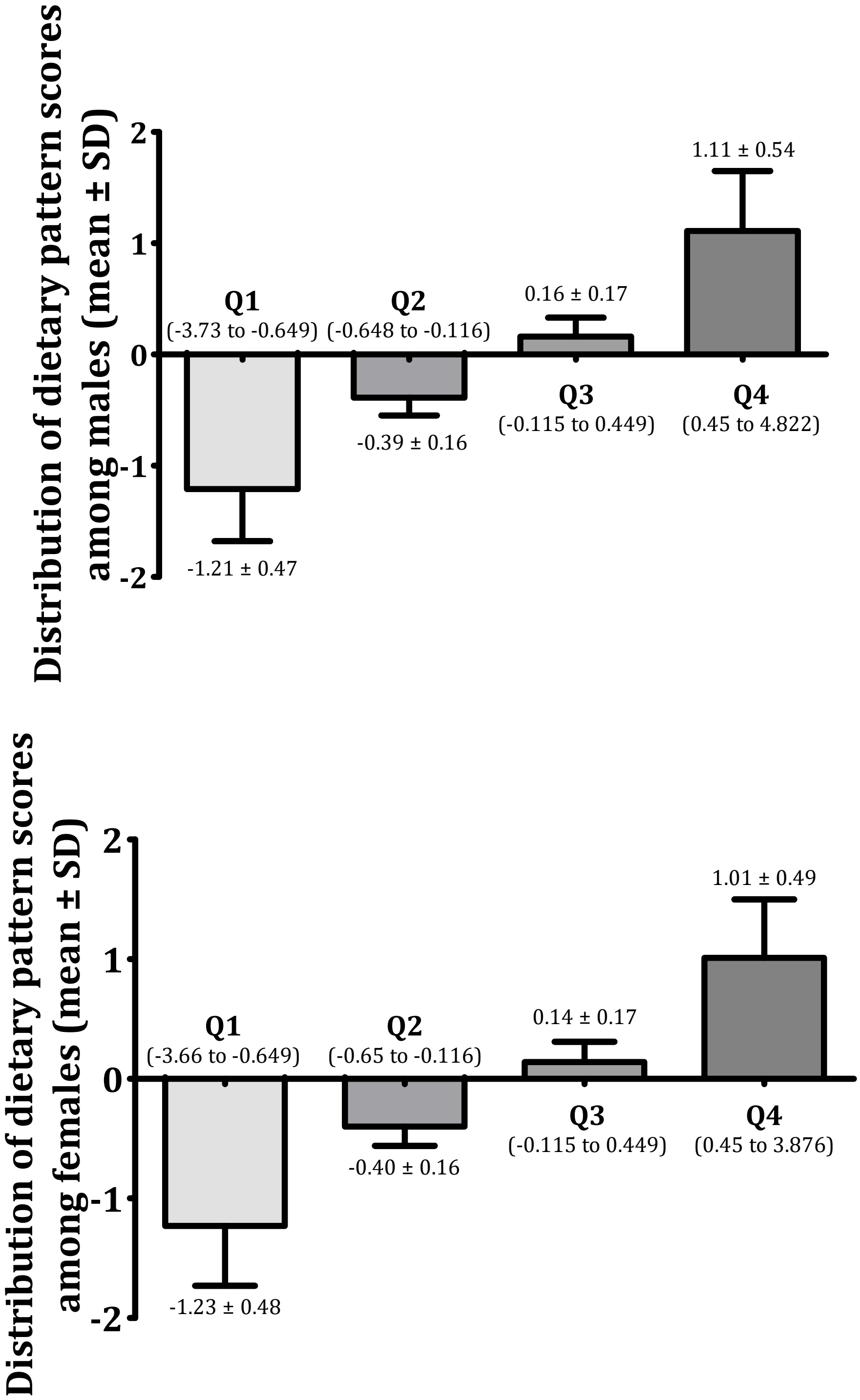

Figure S1. Distribution of dietary pattern scores among males and females.
